# A Novel Blockchain-Based Healthcare System Design and Performance Benchmarking on a Multi-Hosted Testbed

**DOI:** 10.3390/s22093449

**Published:** 2022-04-30

**Authors:** Nihar Ranjan Pradhan, Akhilendra Pratap Singh, Sahil Verma, Kavita, Navneet Kaur, Diptendu Sinha Roy, Jana Shafi, Marcin Wozniak, Muhammad Fazal Ijaz

**Affiliations:** 1 Department of Computer Science and Engineering, National Institute of Technology Meghalaya, Shillong 793003, India; niharpradhan@nitm.ac.in (N.R.P.); akhilendra.singh@nitm.ac.in (A.P.S.); diptendu.sr@nitm.ac.in (D.S.R.); 2 Department of Computer Science and Engineering, Chandigarh University, Mohali 140413, India; sahilverma@ieee.org (S.V.); kavita@ieee.org (K.); navneet.e11384@cumail.in (N.K.); 3 Bio and Health Informatics Research Lab, Chandigarh University, Mohali 140413, India; 4 Machine Learning and Data Science Research Lab, Chandigarh University, Mohali 140413, India; 5 Bio-Intelligence Research Lab, Chandigarh University, Mohali 140413, India; 6 Department of Computer Science, College of Arts and Science, Prince Sattam Bin Abdul Aziz University, Wadi Ad-Dawasir 11991, Saudi Arabia; j.jana@psau.edu.sa; 7 Faculty of Applied Mathematics, Silesian University of Technology, 44-100 Gliwice, Poland; 8 Department of Intelligent Mechatronics Engineering, Sejong University, Seoul 05006, Korea

**Keywords:** Blockchain, Hyperledger Fabric, Gossip protocol, RAFT orderer, Electronic Healthcare Records (EHR)

## Abstract

As a result of the proliferation of digital and network technologies in all facets of modern society, including the healthcare systems, the widespread adoption of Electronic Healthcare Records (EHRs) has become the norm. At the same time, Blockchain has been widely accepted as a potent solution for addressing security issues in any untrusted, distributed, decentralized application and has thus seen a slew of works on Blockchain-enabled EHRs. However, most such prototypes ignore the performance aspects of proposed designs. In this paper, a prototype for a Blockchain-based EHR has been presented that employs smart contracts with Hyperledger Fabric 2.0, which also provides a unified performance analysis with Hyperledger Caliper 0.4.2. The additional contribution of this paper lies in the use of a multi-hosted testbed for the performance analysis in addition to far more realistic Gossip-based traffic scenario analysis with Tcpdump tools. Moreover, the prototype is tested for performance with superior transaction ordering schemes such as Kafka and RAFT, unlike other literature that mostly uses SOLO for the purpose, which accounts for superior fault tolerance. All of these additional unique features make the performance evaluation presented herein much more realistic and hence adds hugely to the credibility of the results obtained. The proposed framework within the multi-host instances continues to behave more successfully with high throughput, low latency, and low utilization of resources for opening, querying, and transferring transactions into a healthcare Blockchain network. The results obtained in various rounds of evaluation demonstrate the superiority of the proposed framework.

## 1. Introduction

Healthcare has always been one of the largest sectors of society dealing with managing patients and their health data [1,2,3]. In the current scenario, data are being stored digitally and are referred to as Electronic Health Records (EHRs) [4,5]. Currently, EHRs are not designed in such a way that they could manage multi-institutional data. Patients’ data are fragmented across different institutions. This makes accessing a patient’s past data difficult. An additional barrier to this is the interoperability between different patient data providers and hospital systems. Due to this, patients and providers face difficulties while initiating the data retrieval process [6,7,8]. While designing a new system to overcome the existing issues, a decentralized database which is constantly updated may present many advantages to the healthcare industry [9,10,11]. For example, different parties may need to access the same information, and if the database is shared, then it becomes easy to access the records. This is where the Blockchain technology seems to be more effective. For example, different involved parties (e.g., general medical specialists [12,13], hospitals [14,15] therapists [16], etc.) could act as a node on the Blockchain network and may share the information among themselves [4,7]. Traditionally, the healthcare records are based on manual ledgers, but with time, these ledgers became inefficient in storing patients’ information due to cumbersome tracking records and increased cost [17,18,19].

Decentralization, security, immutability, and distribution are the key properties of Blockchain [20,21,22]. So, it can address many issues faced in healthcare systems and can provide viable solutions to it. Hence, Blockchain technology can address issues faced in current digital healthcare systems effectively [2,5]. Existing issues such as fragmented patient data and data security of the healthcare may be resolved if new technologies such as Blockchain are used. Blockchain has properties such as data privacy, security and decentralization, which will help to transform existing systems and will make the system more efficient. Blockchain uses a set of access right rules that can share the data among needy users. The rules are nothing but the smart contract that are used in various decentralized application [23,24].

In addition to security of healthcare transaction data, the Blockchain technique depends on two key concepts: consensus and transaction broadcast [25,26]. Consensus among the healthcare [27] peers is achieved by the addition of new blocks in an untrustworthy network by the distributed Byzantine Fault Tolerant (BFT) algorithm. BFT in a public blockchain healthcare network is not sufficient because of the miner who can append ill-formed blocks to the chain and can lead to a threat for Sybil attacks [28,29]. However, a Crash Fault Tolerant (CFT) is sufficient to solve the above issue [1,25]. Moreover, in a permissioned blockchain-based healthcare framework, the trusted membership services and identified participants transactions ordering, called an orderer, have provided a full proof solution by Practical Byzantine Fault Tolerant (PBFT) [30]. HF provides various consensus ordering mechanism such as *SOLO* [1], *Kafka* [19] and *RAFT* [20]. *SOLO* is resilient to BFT and CFT and is not suitable for the healthcare industry, whereas *Kafka* and *RAFT* ordering solutions are most suitable, as it supports distributed fault tolerance [1,25].

The broadcasting of transactions from the source to all other nodes in a Blockchain network relies on the use of Gossip protocol. It spreads the information between nodes in a randomized and probabilistic way, which impacts the transaction traffic and leads to performance bottlenecks. Many studies [2,4] have provided significant solutions scalability and performance of the consensus; however, few studies [3] have paid little attention to the impact of Gossip-based broadcasting in HF [8]. Motivated from the above gap, we designed and implemented a blockchain-based healthcare framework using Raft ordering services that disseminates fair Gossip distribution.

The main contributions of this paper include the following:

- The Blockchain-enabled healthcare framework implementation and prototype design using on-chain and off-chain scheme is presented. *Hyperledger Fabric* is used for enacting smart contracts for client communications. A thorough performance evaluation of this prototype is presented herein.
- Performance analysis of the implemented healthcare prototype on a multi-hosted testbed by employing the Google Cloud Platform (GCP).
- Analysis of network transactions cost and fair optimized traffic owing to *Gossip* protocols resulting in a fair and efficient dissemination. Such an attempt is not yet studied for any Blockchain-based healthcare systems.
- The proposed framework enhances the propagation of blocks to all healthcare peers by 8 times faster than actual implementation while decreasing the network bandwidth and increasing throughput by more than 30%.
- Integrated use of the latest and far more reliable transaction orderer services such as *Kafka* and *RAFT* unlike *SOLO* [1].

To the best of the knowledge of the authors, this paper presents a complete and comprehensive performance study for Blockchain-based healthcare systems with state-of-the-art Raft, multi-host, docker swarm network schemes not yet investigated in the literature. The rest of the paper is organized in the following manner. Section 2 reviews the related work to a Blockchain-based healthcare system and performance benchmarking. The proposed framework and system architecture has been introduced in Section 3. In Section 4, a detailed implementation procedure has been given. Based on the performance benchmarking, Section 5 provides an analysis. Finally, Section 6 concludes the paper with future work.

## 2. Related Work

In this section an overview of related work on blockchain based solutions for EHR system has been presented in Table 1. Jabarulla et al. [17] have proposed a proof-of-concept-based Patient-Centric Image Management (PCIM) system, where a set of access rules called patient-centric access control smart contract is implemented in the Blockchain. The authors have implemented an Ethereum testnet Blockchain with an Inter Planetary File System (IPFS) for storing the medical images and accessing globally through secure hash values. Mazumdar et al. [9] have proposed an anonymous endorsement scheme for Hyperledger fabric called fabrics constrain-sized linkable ring signature (FCsLRS). The suggested scheme analyzed the security and performance of the network with variations of the Rivest Shamir Adleman (RSA) key size. However, Hyperledger supports an Elliptical Curve Digital Signature Algorithm (ECDSA), so the inclusion of RSA for endorsing policy in the EHR system has performance bottlenecks. Stamatellis et al. [13] have designed privacy-preserving healthcare using Hyperledger and implemented it with a proof of concept consensus algorithm, and the results have been measured by performing benchmarking. Pongnumkal et al. [10] have presented a performance analysis between Hyperledger Fabric and the private Ethereum network, where it was found that Hyperledger performs better than Ethereum, which can be expected. The authors conducted the simulation with 10,000 transactions and found that the latency of Ethereum is 8 s, whereas Hyperledger Fabric’s latency is 35 s. Although a comprehensive performance evaluation has been performed, the work does not consider evaluating the network transaction cost and traffic, which are also vital factors. Adroulaki et al. [12] have dealt with a Hyperledger Fabric’s performance evaluation using a single channel, Kafka ordering with *Zookeeper* and *Kafka* broker services. The parameters were implemented by varying the block size, transaction per second, CPU resources, number of peers, and organizations.

The authors in [1] have designed a Blockchain-enabled multi-party healthcare framework using Hyperledger Fabric and Composer. They also designed the access rules for each participant and finally measured the performances using Hyperledger Caliper.

It may be noted that most of the works have used *SOLO* or *Kafka* ordering services for Hyperledger Fabric in a single host system for which the transaction rate is comparatively low. Moreover, most works in the literature have not analyzed in detail the network traffic and transaction-related traffic owing to Gossip and TCP protocol. In addition, the performance of the *RAFT* orderer and its fault tolerance has not been studied to date.

## 3. System Architecture for the Proposed Framework

**Figure 1:**
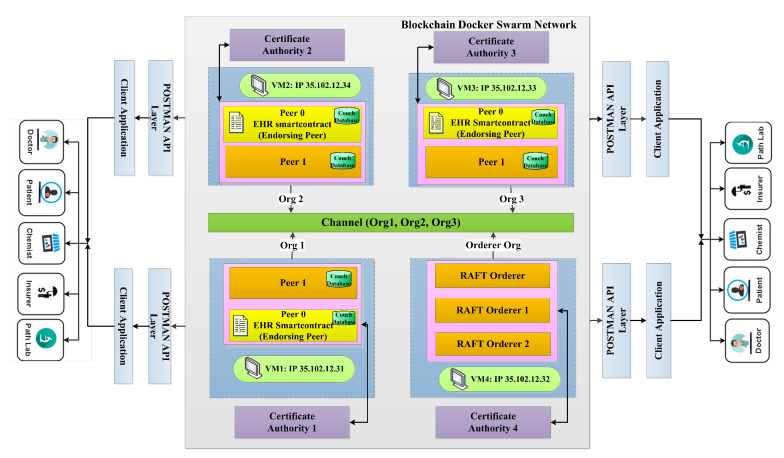
Multi-Host Framework for Blockchain-Enabled Healthcare.

The proposed framework is based on a prototype design, Multi-Host and optimized Gossip Framework for Blockchain-Enabled Healthcare. Figure 1 depicts the proposed framework for privacy preservation in a healthcare process.

### 3.1. Proposed Network Model

**Figure 2:**
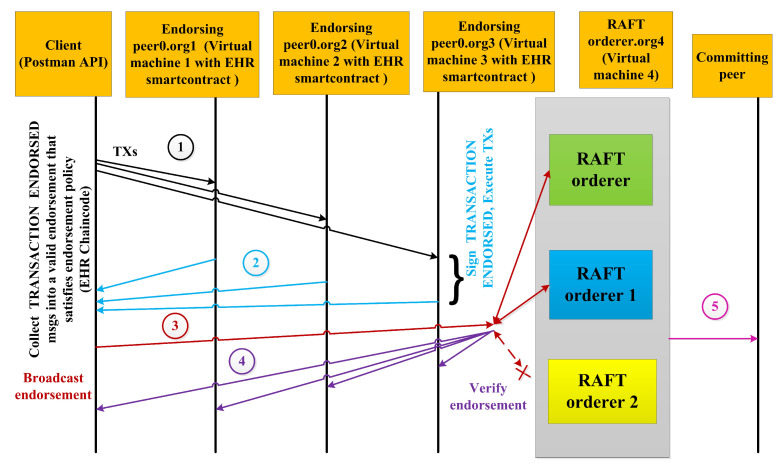
Transaction flow sequence in the proposed system.

**Figure 3:**
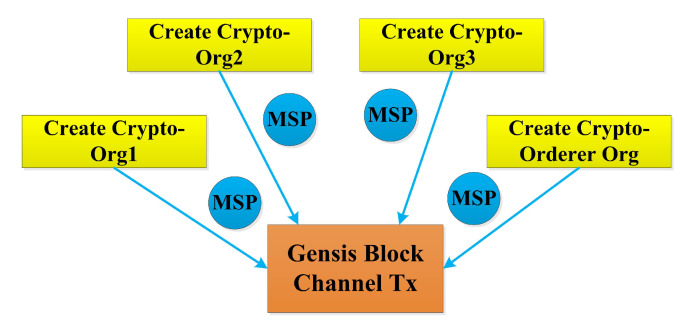
Genesis block creation by crypto materials.

In this section, a Hyperledger Blockchain-based EHR application is proposed for various hosts with an efficient *RAFT* orderer, as depicted in Figure 1. The organizations with multiple peers are proposed in four virtual machines (VMs) in the Google cloud platform. Organization 1 (Org 1) is designed to be hosted on a Virtual Machine (VM) 1, organization 2 (Org 2) on VM 2, Org 3 on VM 3, and orderer services org on VM 4. Different services communicate with each other via the docker swarm network. Dedicated certificate authority for each organization has been designed. Peer 0 is intended to be an endorsing peer, where EHR smart contract resides and peer 1 is an anchor peer. Each peer is proposed to have the current state database as the couch DB. The sequence of our proposed work is the creation of a channel; then, each peer must join the channel, install the EHR chain code, approve the chain code, commit the chain code if it gets sufficient approvals from the organization, invoke the chain code, query the chain code and enable client communication with Postman API. Figure 2 depicts the transaction flow sequence of the proposed framework and the fault-tolerant capability of the RAFT orderer. It shows that if any one of the orderers fails out of three orderers, still, the transaction endorsing and committing takes place. Although there are several ordering services to handle transactions and configurations, a RAFT orderer has been proposed for the following reasons. It is an efficient crash fault-tolerant (CFT) algorithm that follows the leader and follower concept, where the follower follows the decisions made by the leader node elected per channel. It is designed to handle the distributed applications. The Kafka also follows the leader and follower principle but utilizes Zookeeper ensembles and a broker, which creates an overhead with respect to transaction latency and throughput. SOLO ordering services are only used for testing purposes, not for production, as its designed principle is based on a single organization with a single peer. RAFT provides a strategy for high availability for ordering services because of its endorsement policy of majority voting.The Genesis block was offered and executed after creating the crypto materials shown in Figure 3. The proposed framework has been evaluated using Caliper, and network traffics have been analyzed for the Gossip protocol.

### 3.2. Proposed Transactions

The transaction authentication in the proposed framework is divided into two parts, namely, off chain and on chain as shown in Algorithm 1. Off-chain in Algorithm 1 deals with the doctor, patient, and pharmacy validator nodes in the network by issuing them access rights and identity privacy. These participants broadcast their appointment, prescription, and buying medicine data details by signing with their individual private keys, as shown in (1)–(3). The integration of messages are named as I1, I2, and I3. The RAFT orderer nodes O1 and O2 sign the transaction and add a timestamp if it finds a match, as shown in (4) and (5). After this, the on-chain permissioning starts. The on-chain permissioning deals with the network that verifies and validates the transaction in their respective peer nodes and submit transactions by adding their keys. The actual patient-related transaction, R, is calculated by using Keccak and has functions as shown in (6). Finally, the Blockchain transaction record, TR, is added to the ledger by integrating (I4) the timestamp with the patient transaction hash record, as shown in (7). The participants, i.e., patient, doctor, and chemist authentication is designed in the framework by specifying the access rights and unique identity. The patients, doctors, and chemists register their diseases, specialization, and chemist details by signing with their private keys as depicted in Equations (1)–(3).

(1) $$Patient_{Data}=I_{1}\left(P_{Id},P_{Addr},P_{Diseases},P_{PK},P_{AS},P_{BM}\right)$$

$P_{AS}$—Appointment status of patient

$P_{BM}$—Buy medicine by patient

(2) $$Doctor_{Data}=I_{2}\left(D_{Id},D_{Addr},D_{AS},D_{PK},D_{PId},D_{Specializations}\right)$$

$D_{AS}$—Appointment status of doctor

$D_{PID}$—Prescription ID suggested by doctor

$D_{PK}$—Private key of the doctor

(3) $$Chemist_{Data}=I_{3}\left(C_{Id},C_{Addr},C_{SM},C_{BillId},D_{PK}\right)$$

$C_{SM}$—Sell medicine by chemist

Then, with the Raft orderer, the transactions are time stamped, as shown in Equations (4) and (5).

(4) $$O1=\left(D_{PId},P_{Id},TS1\right)\;$$

(5) $$O2=\left(C_{SM},P_{BM},TS2\right)\;$$

The Blockchain transaction (*TX*) only records the orderer details along with the hash value of participants ID, as shown in Equations (6) and (7). Deploying any EHR application on a single host machine with multiple peers, organizations, and orderers does not necessarily constitute a decentralized application. The network model, participants, assets, and transactions have been discussed to justify the applicability of the proposed framework.

(6) $$R=Keccak256hash\left(P_{Id},D_{Id},C_{Id}\right)\;$$

(7) $$\mathrm{TX}=I_{4}\left(O1,O2,R\right)\;$$

**Algorithm 1:** Algorithm for participant creating, initializing and querying healthcare records (buying, selling medicine and appointment status matching)

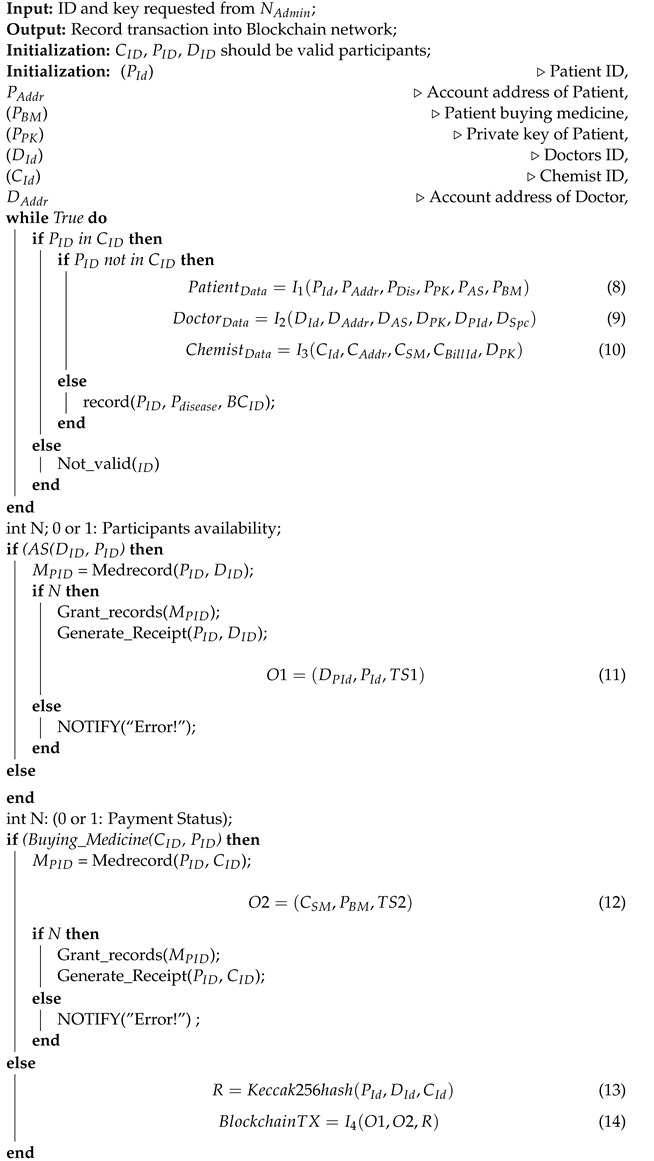



### 3.3. Proposed Participants, and Assets

For the proposed Blockchain-based multi-hosting healthcare framework, the basic entities are participants, assets, transactions, and control logic designed through Hyperledger Fabric 2.0. These entities are designed to perform some particular functionality governed by a set of rules called EHR smart contracts.

- Participants: The individual entities in healthcare organizations such as doctors, patients, chemists, insurers, and path lab are called participants.
- Assets: Doctors as a participant create a prescription asset for the patients. The patient as a participant pays a cash or coin asset for buying medicine, consulting doctors fees, and collecting path lab testing reports. The chemist, as a participant, generates a receipt for the sold drugs. Similarly, the path lab as a participant generates lab test reports as an asset for patients. Assets are tangible or intangible.
- Transactions: A transaction in a healthcare system is a read, write, or update operation that follows the sequence such as instantiated, invoked, endorsed, validated, ordered, committed, and finally broadcasted to the intended users. In our proposed work, three transactions are considered as creating an EHR, initializing the ledger, and querying a healthcare system.

## 4. Implementation

This section is the deployment and implementation of the proposed framework.

### 4.1. Experimental Setup

Instead of working with real systems and real hardware, this approach simulates the interaction of different components, without necessarily imitating the complete network stack. This provides a completely controlled and reproducible environment in which the experiments are conducted. Because there is no direct dependency on hardware and real networks, it is easier to scale the size of the network by varying the total number of transactions, rate control, number of virtual machines, blocksize, number of rounds, and so forth. The possibility to keep the complete evaluation on a single machine provides easier debugging and even time manipulation, which allows simulating large loads on the network. These aspects would be more difficult on other evaluation platforms and hardware because of their distributed architecture. There are different kinds of software platforms that can be used to evaluate network technologies such as Blockchain and Distributed Ledger Technologies (DLTs). We have used Hyperledger Caliper. Caliper is a benchmarking tool for Blockchain. It supports some Hyperledger Fabric Blockchain implementations and is built to be easily extensible to other technologies. In this paper, this tool is utilized to define, generate, and execute the workloads. Additionally, it also provides the on-chain metrics for evaluation. The EHR smart contract is written in JavaScript and deployed in the source file.

### 4.2. Deployment of Virtual Machines and Other Prerequisites

Four numbers of virtual machines have been deployed on the GCP. Fabric 2.0 has been installed in order to perform multi-hosts for all the virtual machines by using Secure File Transfer Protocol (SFTP). The setting of virtual machine 1 has been carried out and cloned up for machines 2, 3, and 4. The host has been added with IP 35.102.12.31 and connected remotely using visual studio code. The requirements and specification of our proposed network has been shown in Table 2. The Genesis block has been created by configuring the *configtx.yaml* file. Similarly, the artifacts have been created by configuring the *create-artifacts.sh* scripting file. In the *src* file, the EHR smart contract has been loaded. This EHR smart contract contains different methods to invoke and query transactions. For VM 1, all the necessary service files related to Org 1 such as API 2.0 for accessing the application, channel artifacts for secure communication, certificate with CA for authorization, *base.yaml* for peer configuration, *deployChaincode.sh* for EHR chain code deploy, *docker-compose* file for Org 1 services, environment variables for peer0, peer1, CouchDB 0, and CouchDB 1 and a command-line interface (CLI) container have been implemented. The same procedure is applied for Org 2, Org 3, and the orderer. For virtual machines 2, 3, and 4, it does not require a CLI container. Similarly, VM 4 does not require API as it is having multiple orderers. The MSP values of all these organizations is created for the Genesis block and channel. Individual certificate authority has been implemented to complete and sign the certificate for all the participants in the organization. In order to run the RAFT orderer, the genesis block file has been created.

### 4.3. Crypto Materials for Org 1, Org 2, Org 3 and RAFT Orderer

The environmental variables are set in *docker-compose.yaml* so that it can export the services to the 7054 port number. The instances are started; thereby, the certificate authority 1 (CA1) creates the own public and private key and self signs the certificate. The command *docker-compose up-d* was executed to create a fabric CA materials. The key-store inside the generated CA materials contains the private key. The create certificate script file contains a method named *createcertificateOrg 1( )*, where it enrolls the admin identity running on the 7054 port, which is the host-provided TLS certificate to communicate with Org 1. Finally, the node organization unit contains the nodes such as the peer, admin, client, and orderer. While registering, the peer’s CA details, ID name, password, TLS certificate have been provided. The same process was followed by other peers, users, and admins. To create the peer 0 membership service provider (MSP), the peers, users, and admins are enrolled. The peer TLS certificate has been generated, which is used to communicate between two peers of the same organization or with a different organization. Running the script *./create-certificate-with-ca.sh* creates a crypto configuration folder, and all the certificates are generated. All the node organization unit materials related to CA, MSP, peers, TLSca, and users for organization 1 have been received. In the same way, crypto materials are created for organizations 2 and 3, and the services are exported to port numbers 8054 and 10054, respectively.

### 4.4. Creating Docker Swarm Network

Services are running on different virtual machines. However, to communicate with each other, the docker swarm network has been installed. It has created a network across these virtual machines, and they shared with each other. All the virtual machines are connected through SSH and the respective IP address through console mode. The IP address of all the virtual machines is given in Table 1. To install the docker swarm network, the command, as shown in Listing 1, has been executed on virtual machine 1. To add other virtual machines as a manager into the docker swarm network, the commands are executed as shown in Listing 1. It generates a token value which runs in other virtual machines with their respective IP address. Virtual machine 1 contains the docker network with artifacts. The network artifacts were available in other virtual machines by executing the command ’docker network create attachable’.

**Listing 1:** Creating Docker Swarm Network.

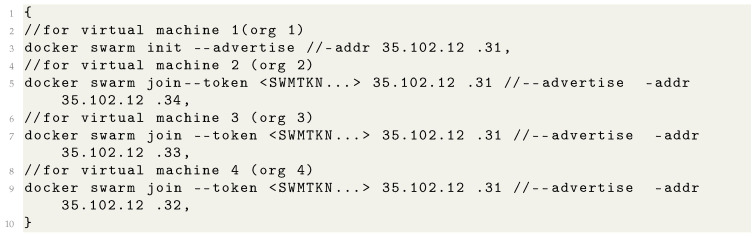

### 4.5. Creating Channel Artifacts

To configure channel artifacts, three scripting files, i.e., *configtx.yaml*, *create-artifacts.sh*, and *crypto-config.yaml* files are being executed. To create genesis block and channel transaction files, the MSP of a particular organization is fetched. The MSP directory path of the organizations and orderer has been provided correctly inside the *configtx.yaml* file. The policies are customized for reading, writing, and endorsements by the majority out of three organizations; at least two organizations must approve, as shown in Listing 2. *Genesis.block*, *mychannel.tx*, *Org 1 MSPanchors.tx*, *Org 2 MSPanchors.tx*, *Org 3 MSPanchors.tx*, system channel, and application channels are created while running the artifacts scripting file. All the crypto materials are added to their virtual machines. To up the services across the network, all the docker containers are executed. Services for peer0, peer1, CLI, couchdb1, and couchdb2 of the respective organization and RAFT orderer, orderer 2, and orderer 3 have been started. These services run inside a container, the communication was started with each other, and the same has been verified in the docker log file.

**Listing 2:** EHR Chaincode Approvals Endorsement Policy.

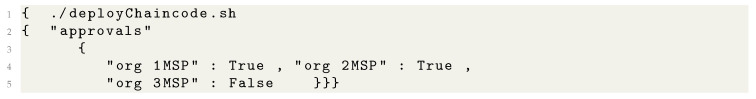

### 4.6. Creating and Joining Channel

The peer channel creates an IP address: the 7050 channel name command is executed to create the channel, but although an orderer is running in virtual machine 4, the IP address was provided instead of the local host. Similarly, running *create channel.sh*, a channel was created, and my channel.block has been generated to connect peer 0 and peer 1 of organization 1. Anchor peers are updated by running the same script file. For organization 2, creating a channel is not required. Fetching and joining the channel is done by running the *joinchannel.sh* file. Bringing the latest configuration block and joining the channel occurred by providing the IP address of virtual machine 2 and orderer properly with respect to peer 0 and peer 1. The same procedure is applied to virtual machine 3.

### 4.7. EHR Chaincode Deployment

The EHR smart contract is written in JavaScript and deployed in the source file. There are many methods defined inside the EHR file. However, three functions are used such as *createEHR( )*, *initledger( )* and *queryEHR( )*. *CreateEHR( )* takes five arguments such as patients name, patient ID, disease name, consulting doctor ID, and medicine suggested. *QueryEHR( )* takes one argument, i.e., patient ID through which the data retrieval takes place from the blockchain network and sending it to the client. The Init function is used for instantiating the chain code, and the invoking method is used for invoking the contract.

### 4.8. Install, Approve, Commit, and Invoke EHR Chain Code

The chain code dependency is executed by setting the preset-up method inside the deploy chain code scripting file. Before installation of the chain code, it is packaged in the tar file. Installing the chain code has been carried out in endorsing peers. Peer 0 of all organizations is set to endorsing peer; that is why the installation of EHR chain code on peer 0 will return the console’s response. The chain code was fetched in order to determine whether the chain code was appropriately installed or not. Then the query method was executed. For the chain code approval policy of majority organizations, the approval for any two organizations is conducted, as shown in Listing 2. To cross-check the approvals, a check commit has been executed, resulting in true or false from various organizations.

### 4.9. Commit Chain Code and Invoke Transaction

Committing the chain code is performed by exporting the peer address, and response is received from all the peers with status, as shown in Listing 3. Similarly, Invoking a transaction is done inside the CLI container bash file, which returns a status ID of success or failures, as shown in Listing 4. The docker shows the EHR chain code inside the container. The query committed on-chain code is found displaying an executed EHR chain code, endorsing system chain code (ESCC), validating system chain code (VSCC), and approvals from organizations. The creation EHR method with a patient ID was invoked successfully, and verification is performed through Faux-ton and the IP address of the virtual machine 1.

**Listing 3:** EHR Chaincode Invoke.

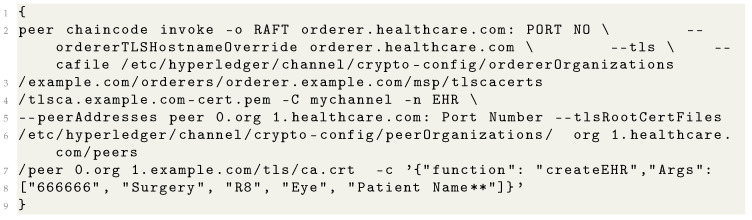

**Listing 4:** EHR Chaincode Query.

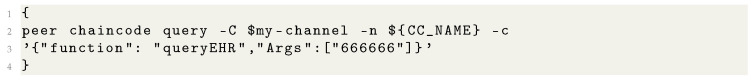

## 5. Performance Analysis and Discussion

**Figure 4:**
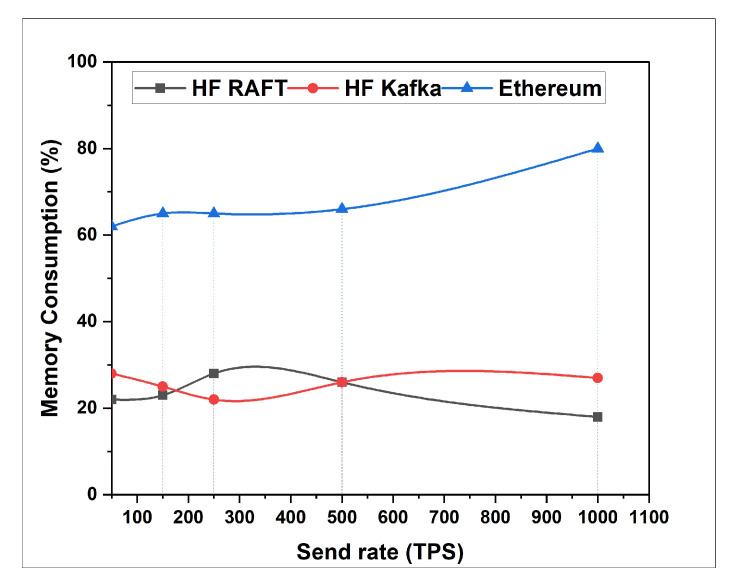
Memory Consumption for Open.

**Figure 5:**
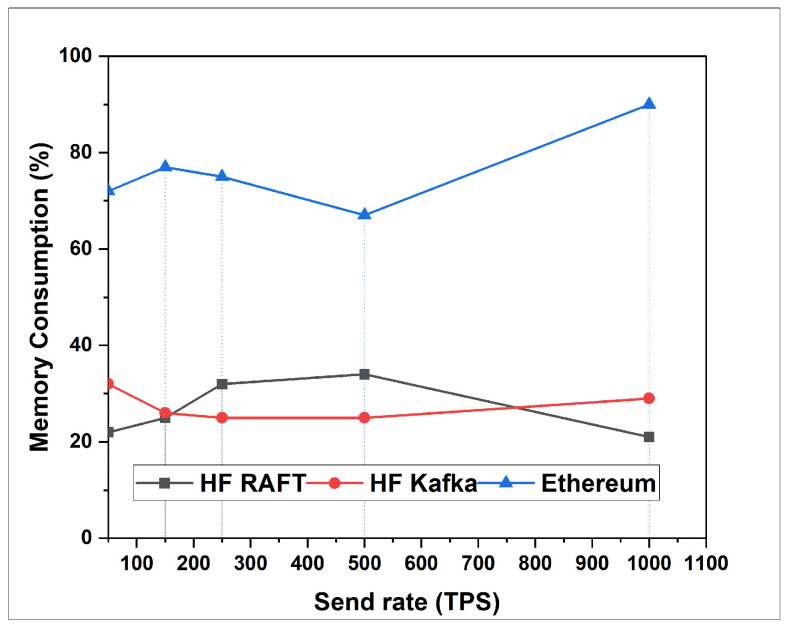
Memory Consumption for Transfer.

**Figure 6:**
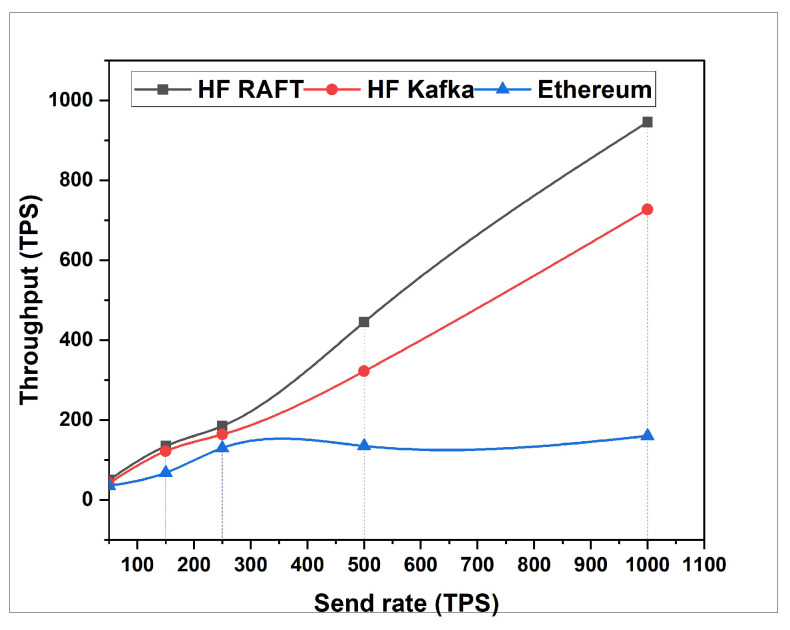
Throughput for Open.

**Figure 7:**
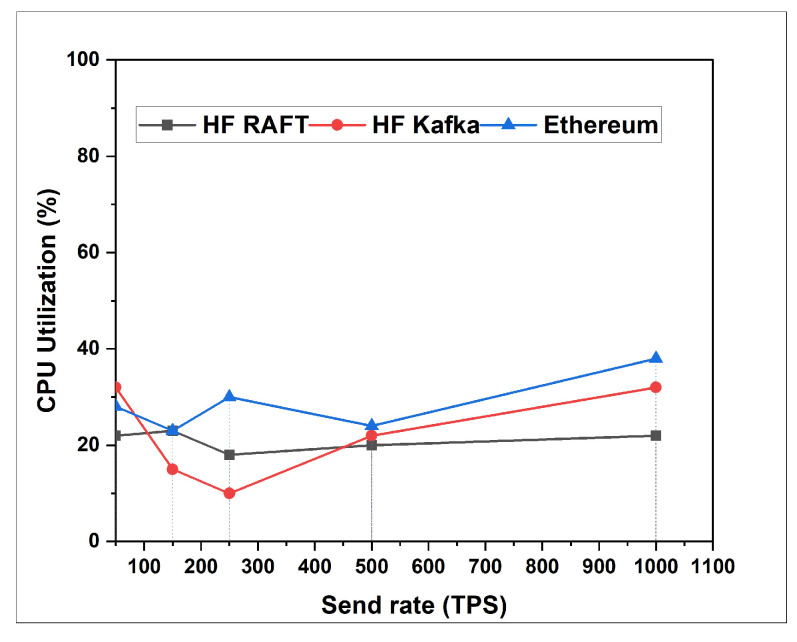
CPU Utilization for Open.

**Figure 8:**
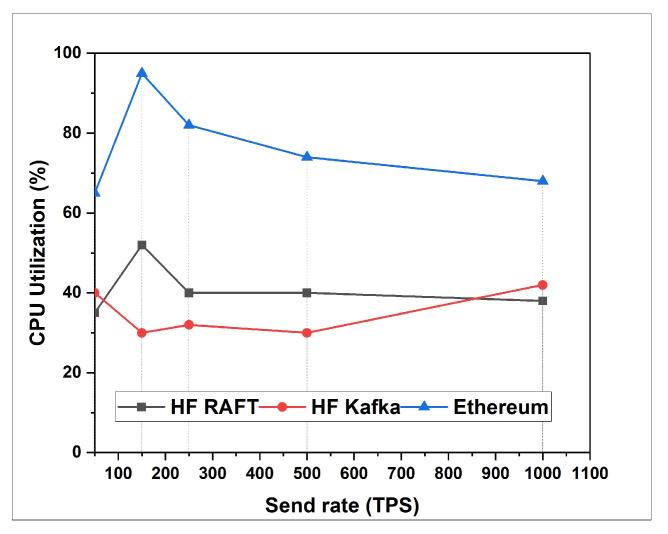
CPU Utilization for Transfer.

**Figure 9:**
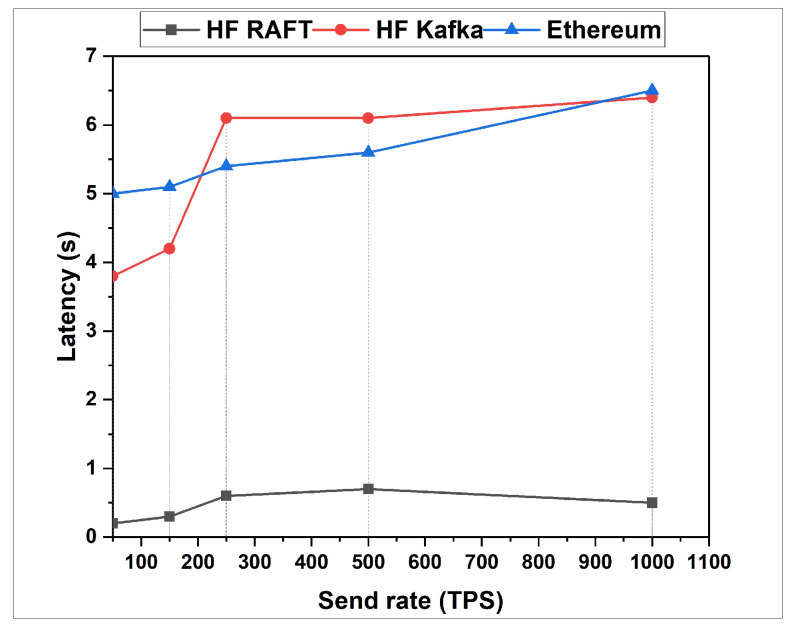
Latency for Open.

In this section, the network resource cost of transactions and traffic inside the fabric multi-host network has been analyzed. Performance comparisons are presented for a Blockchain network with RAFT orderer. Figure 4, Figure 5, Figure 6, Figure 7, Figure 8 and Figure 9 depict the performance benchmarking of different blockchain platform such as Hyperledger Fabric RAFT, Kafka and Ethereum with respect to throughput, memory consumption, latency, and CPU utilization. In all case we found that the proposed framework works fine in terms of low latency, high throughput and low memory and CPU utilization.

### 5.1. Measurement of GOSSIP Traffic

**Figure 10:**
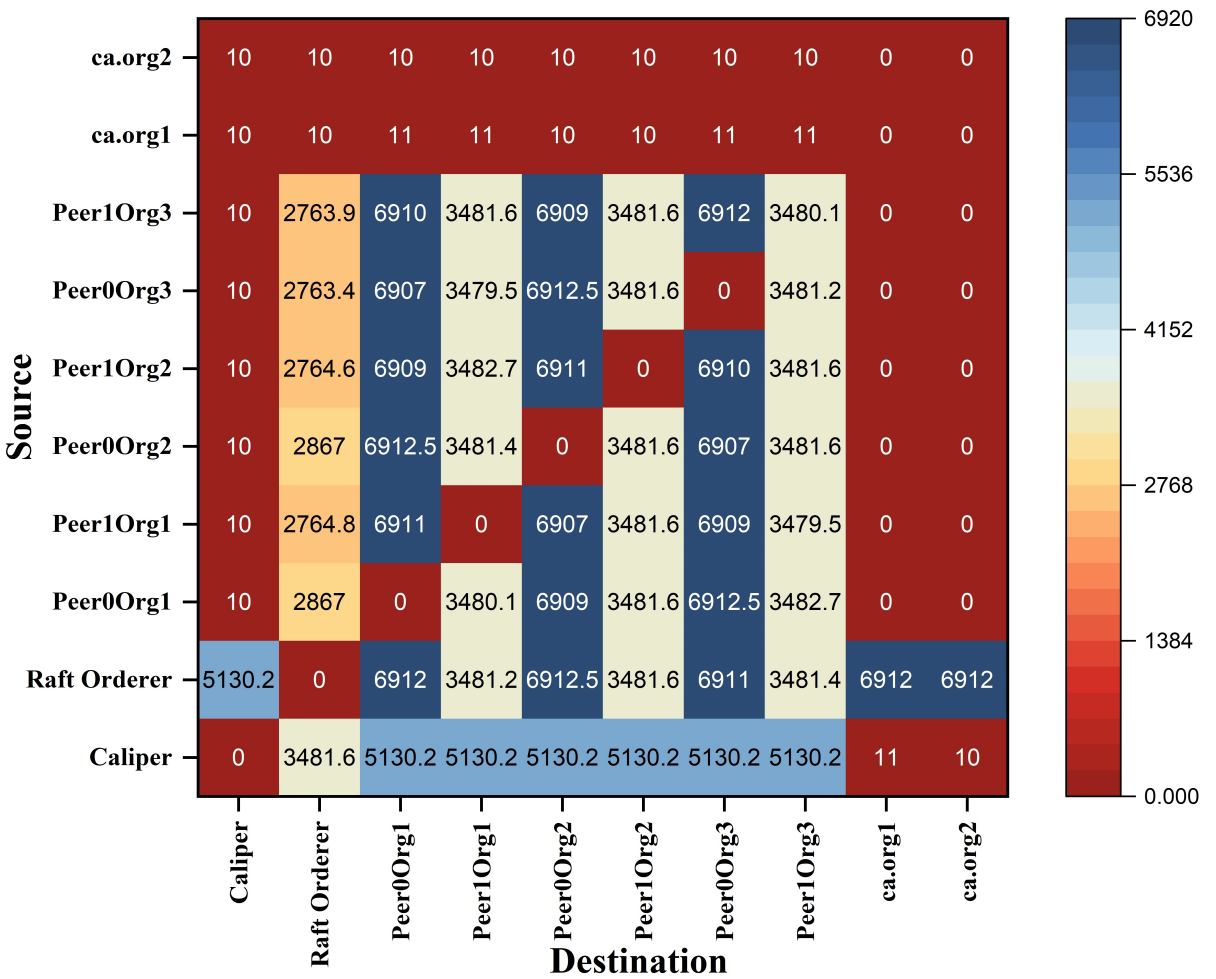
Heatmap of Transaction Traffic Gossip (SOLO).

**Figure 11:**
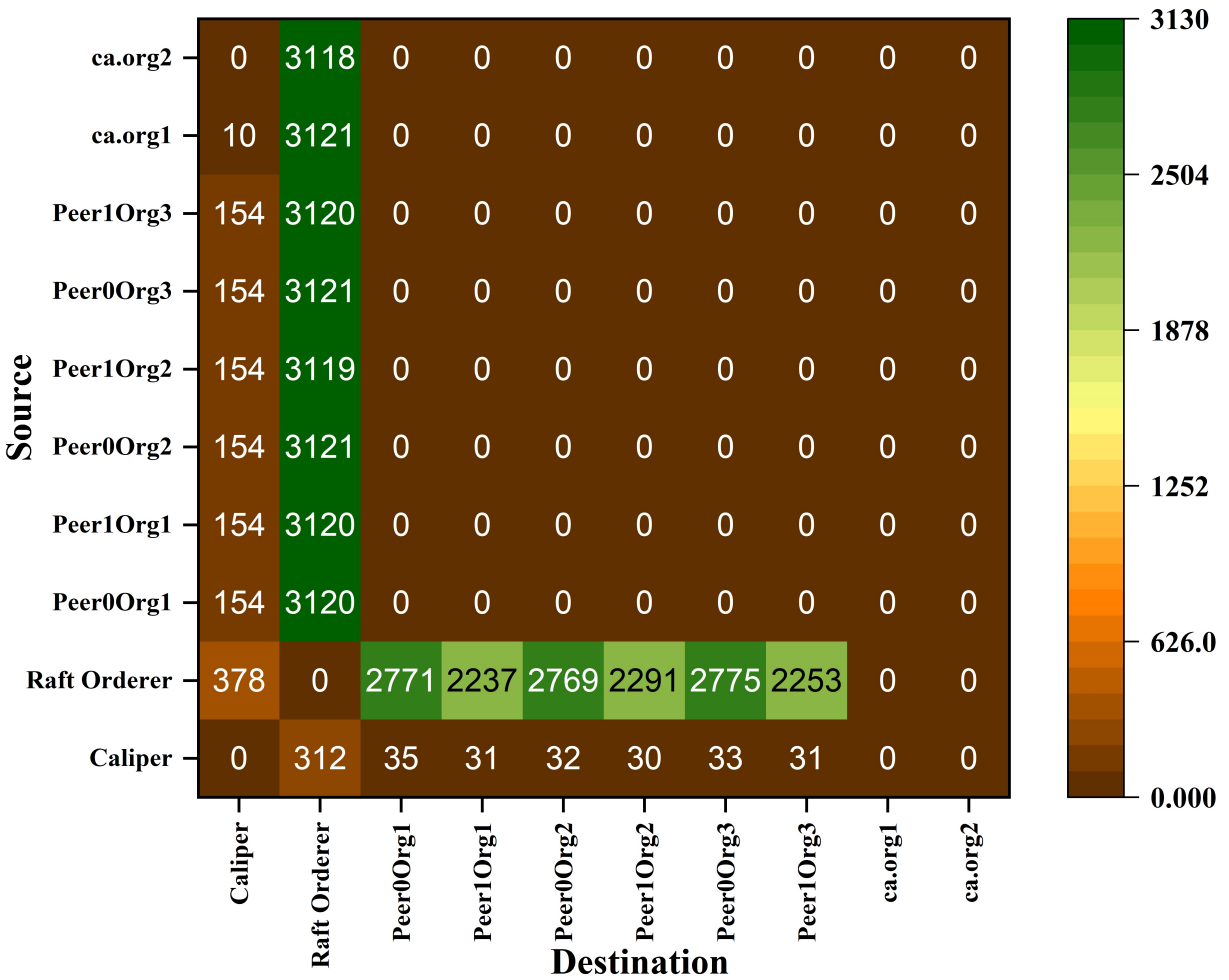
Heatmap of Transaction Traffic Gossip for Proposed Framework (RAFT).

**Figure 12:**
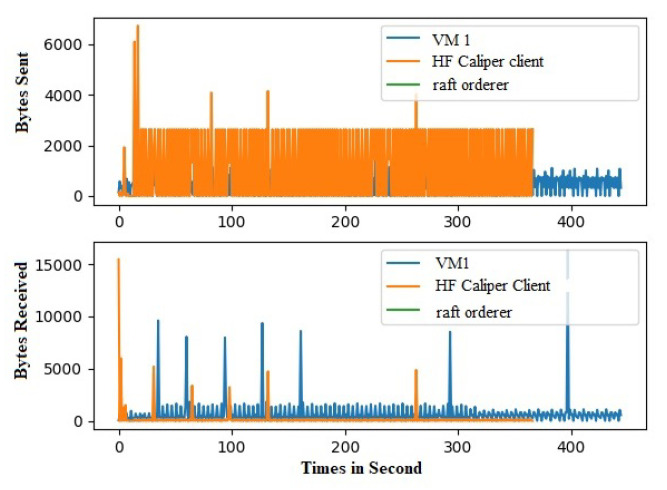
Transaction Traffic for VM 1.

**Figure 13:**
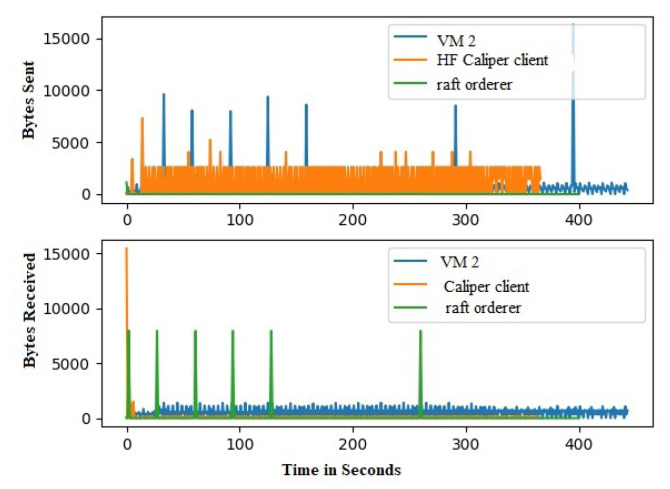
Transaction Traffic for VM 2.

**Figure 14:**
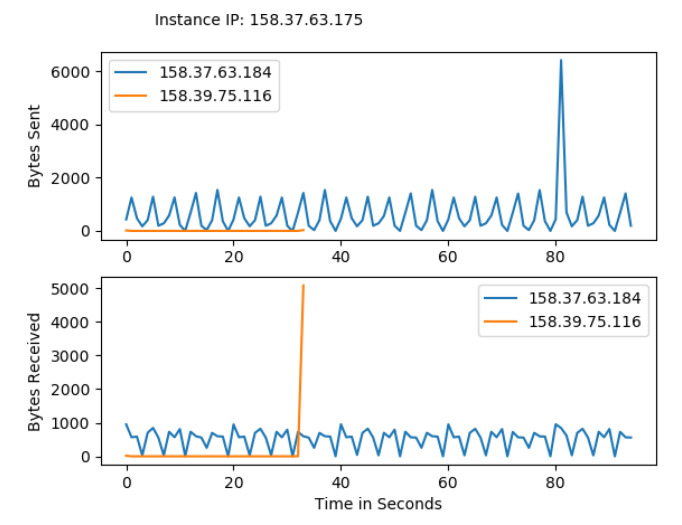
Transaction Traffic for VM 3.

**Figure 15:**
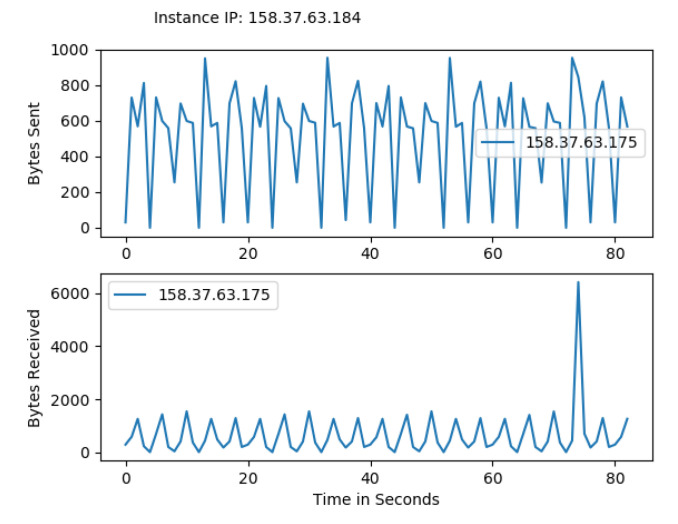
Transaction Traffic for RAFT orderer.

Hyperledger Fabric 2.0 generates a background network while communicating from one peer to other peers in the network. Without measurement of Gossip traffic, it is tough to measure the other background traffic associated with transaction and block distribution. This work performs a network traffic analysis between all the peers, organizations, and virtual machines during an ideal period of Fabric 2.0. So that it prevents the background traffic from interfering with other traffic, this work has not used Hyperledger Caliper during this measurement. Through tcpdump, the transnational traffic has been measured by gathering the PCAP files. The Fabric 2.0 network takes a little time to settle, and the network was started and waited for some time. The start of the fabric network generates extra traffic. A delay has been introduced in order to avoid the mixing of transnational traffic with other traffic. The results are with a delay of two minutes. Although most of the Fabric network communication utilizes the TCP protocol, we used a packet filter to capture the SSH traffic and the peers traffic. All the individual pcap files are collected along with merged pcap files, which have been trimmed to force the virtual instances to report data from equal time spans. The Wireshark CLI tool is used to merge and trim the files. A merged pcap file was analyzed, and Gossip protocol was calculated. We found that the transnational traffic is more intra-organizational than inter-organizational. As noticed, the data transmission among the caliper client and other organizations, peers, and orderers is exceptionally high. This work discovered that most of the traffic is not created by transactions. This work filtered out this traffic to obtain results from the transaction alone. Figure 10 and Figure 11 represents the heatmap of transaction traffic for SOLO and RAFT ordering.Figure 12, Figure 13, Figure 14 and Figure 15 depict the virtual machines communication with respect to caliper client and RAFT orderer.

### 5.2. Performance Measurement Using Caliper

**Figure 16:**
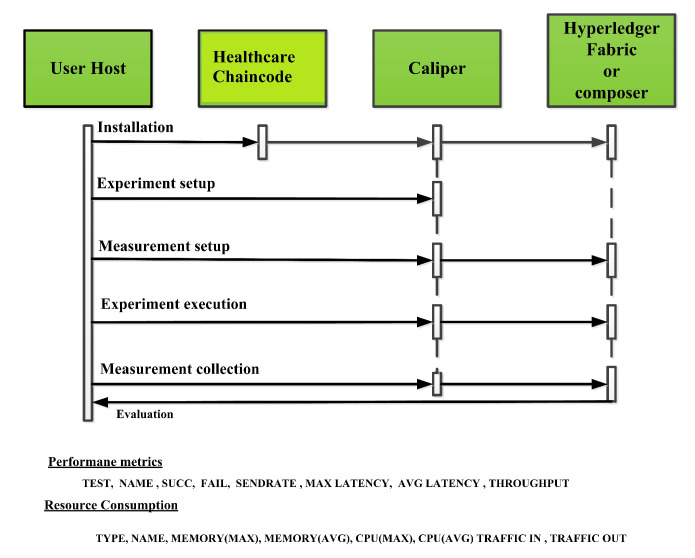
Caliper benchmarking process.

In this section, the evaluation of the performance metrics are evaluated and monitored; then, we compared the performance of the proposed framework based system with different types of Blockchain-based healthcare systems. We consider Hyperledger Fabric Blockchain platforms and two types of consensus algorithms (i.e., RAFT and Kafka) for the performance evaluation. We also used Hyperledger Caliper benchmarking process as shown in Figure 16 to see the performance behavior of our proposed healthcare framework by giving a load of 1000 transactions with two rounds (i.e., open and query) and three sub-rounds with varied rate control (i.e., 50, 100, and 150). We open a pre-defined JavaScript file to open and initialize the states. Similarly, queryState() is used to read state from the ledger (in Fabric implemented by invoking but not committing a chain code). The performance metrics used for comparison are block time, latency, throughput, transaction success rate, fail rate, memory, and CPU utilization. The performance result indicates that the proposed RAFT-based system has better performance than other consensus mechanism. Table 2 in the paper has additionally been added to justify the work proposed [1].

Here, the equations for Latency, throughput and disc read writes are given.

(15) TL=((CT)∗(NT))−(ST)

(16) $$TT=\frac{TCT}{TTS}-\left(NCN\right)$$

(17) RL=(RR−ST)

(18) RT=(RO−TT)

where *TL*: Transaction Latency, *CT*: Confirmation Time, *NT*: Network Threshold, *ST*: Submit Time for transaction *TT*: Transaction Throughput, *RL*: Read Latency, and *WL*: Write Latency.

From Table 3, we found that the proposed framework is more successful in making transactions, i.e., open. Out of 1000 transactions each, RAFT could perform 946 transactions, whereas Kafka completed only 727 transactions. Comparing the throughput, RAFT has a higher throughput compared to that of Kafka. Querying a transaction in a block in RAFT and Kafka has the same probability of success, i.e., 100 %. However, the throughput of Kafka is measured high as compared to that of RAFT. The reason behind the higher throughput of Kafka is due to its batch time and batch size. Transferring a transaction value from one account to another also has an equal probability of success, whereas considering RAFT’s throughput is faster than that of Kafka. RAFT’s success is RAFT’s consenter invoking the transaction directly from the orderer nodes, whereas Kafka depends on the Kafka brokers and Zookeeper services. After the first and second rounds for a multi-host Raft orderer, the optimized resource consumption values are given in Table 4 and Table 5.

## 6. Conclusions

Deploying a Blockchain network in a single host with multiple organizations, peers, and channels logically looks simple at the surface, yet such simulation attempts cannot capture the essence of real decentralized scenarios for distributed applications such as next-generation Blockchain-based healthcare systems. This paper presents a novel multi-host, multi-organization, on-chain and off-chain scheme for storing patient data and multiple peer-based frameworks for a Blockchain-enabled healthcare system that addresses the issues of data availability, data privacy, and security using a real multi-host testbed, namely GCP. The paper presents a detailed implementation of the healthcare system prototype with Hyperledger Fabric 2.0, docker swarm network, and Postman API for client interaction, Caliper for performance analysis, *tcpdump* for realistic network traffic generation, orderer for *RAFT* and *Kafka*, etc. A comparison of *Kafka* and *RAFT* orderer services was also presented, and *RAFT* was found to be better equipped for the open, query, and client-side transfer operations. In the future, the authors aim to work on cross-chain code communication, discovery services, and PBFT consensus-based healthcare systems. In addition, the authors aim to extend the work with other Hyperledger Frameworks, i.e., Hyperledger Sawtooth, Besu, etc., in order to deal with better performances in terms of throughput, scalability, latency, and fault tolerance. 

## Figures and Tables

**Table 1:** Related work on blockchain-based approaches for the EHR system.

References	Year	Objective	Performance	Limitation	Performance Evaluation
Azaria et al. [26]	2016	MedRec: Ethereum based permission less mode of operation	It slows down network computing over the time and makes the network less transparent	Full transparency	No
Shen et al. [27]	2019	MedChain: patient centric healthcare by providing the privacy preserving mechanism for healthcare data	Only calculated average response time, throughput and average message time based on Ethereum	Full transparency	Partially
Gorenflo et al. [28]	2018	To scale a blockchain network using Hyperledger Fabric	Demonstrable capability of blockchain network	Increased computing power needed	No
Sun et al. [29]	2018	To propose a decentralizing attribute based signature using blockchain	Verifiable secure sharing of large-scale and distributed EHR	Attribute certificates, storage capacity	Partially
Chen et al. [30]	2019	To design a searchable encryption for EHR using blockchain	Security analysis with searchable encryption algorithm	Scalability	No
Singh et al. [1]	2020	To design and propose an efficient blockchain based EHR system using HF and SOLO ordering services	EHR with smart contract, achieves performance optimization using Caliper	No fault tolerance capacity of the network	Yes
Proposed Approach	2022	To design and propose an efficient blockchain based EHR sharing with HF and RAFT ordering services with on-chain and off-chain storing scheme	Transaction traffic analysis and performance optimization using Caliper	Fault tolerance	Yes

**Table 2:** Requirements and specification of proposed EHR Blockchain network.

Requirements	Specification
Operating System	Ubuntu Linux 18.04 (8 GB RAM)(64 bit)
Virtual machine 1 (35.102.12 .31)	Ubuntu Linux 18.04 (2core, 8 GB RAM, 30 GB memory, 64 bit)
Virtual machine 2 (35.102.12 .34)	Ubuntu Linux 18.04 (2core, 4 GB RAM, 30 GB memory, 64 bit)
Virtual machine 3 (35.102.12 .33)	Ubuntu Linux 18.04 (2core, 4 GB RAM, 30 GB memory, 64 bit)
Virtual machine 4 (35.102.12 .32)	Ubuntu Linux 18.04 (2core, 4 GB RAM, 30 GB memory, 64 bit)
cURL Tool	Version 7.74.0
Docker engine	Version 17.06.2
Docker Composer	Version 1.14
Javascript	1.8.5
Node JS	Version 10.21
NPM	Version 6.14.4
Hyperledger Fabric	2.0.1
VS Code	1.49.1
Docker Swarm Network	12.06
Postman API	v7.333.0
Hyperledger Caliper	v0.4.2
Fauxton Apache couch DB	version 6.1

**Table 3:** Efficacy of the proposed multi-host, RAFT framework with 1000 transactions.

Name	No. of TXs	Succ	Send Rate (TPS)	Avg Latency (s)	Throughput
RAFT (Open)	1000	946	50, 150, 250	0.2, 0.3, 0.5	50, 135, 185
Kafka (Open)	1000	727	50, 150, 250	3.8, 4.2, 6.1	42, 122, 164
RAFT (Query)	1000	1000	50, 150, 250	0.12, 3.63, 7.62	66, 77, 87
Kafka (Query)	1000	1000	50, 150, 250	4.12, 5.86, 8.11	67, 84, 105
RAFT (Transfer)	1000	1000	50, 150, 250	0.2, 0.3, 1.3	45, 61, 92
Kafka (Transfer)	1000	1000	50, 150, 250	3.2, 3.3, 4.6	32, 53, 84

**Table 4:** Resource consumption after first round (RAFT).

Type	Name	Memory (Max MB)	Memory (Avg MB)	CPU (Max)	CPU (Avg)	Traffic In	Traffic Out	Disc Write
Docker	peer 0.org 1.35.102.12.31	403.8	389.3	5.23%	3.13%	3.3	1.9	18.3
Docker	peer 1.org 1.35.102.12.31	512.9	406.4	4.52%	3.7 %	3.3	1.86	18.3
Docker	peer 0.org 2.35.102.12.34	205.3	200.3	5.16%	3.03%	3.3	1.7	17.3
Docker	peer1.org2.35.102.12.34	201.3	200.3	4.57%	3.23%	3.3	1.77	17.3
Docker	peer 0.org 3.35.102.12.33	203.9	200.3	5.64%	3.01%	3.3	1.81	16.3
Docker	peer 1.org 3.35.102.12.33	146.6	119.3	4.28%	3.04%	3.3	2.0	16.3
Docker	RAFTorderer.35.102.12.32	19.6	18.0	1.01%	0.26%	2.3	4.5	8.0
Docker	ca.org 1.35.102.12.31	8.3	7.6	0.13%	0.00%	1.5	0	0
Docker	ca.org 2.35.102.12.34	8.3	7.6	0.13%	0.00%	1.5	0 B	0 B
Docker	ca.org 3.35.102.12.33	8.6	7.8	0.29%	0.00%	1.4	0	0

**Table 5:** Resource consumption after second round (RAFT).

Type	Name	Memory (Max MB)	Memory (Avg MB)	CPU (Max)	CPU (Avg)	Traffic In	Traffic Out	Disc Write
Docker	peer 0.org 1.35.102.12.31	405.7	138.0	6.47%	3.16%	6.6	3.61	34.5
Docker	peer 1.org 1.35.102.12.31	405.7	125.0	6.19%	3.16%	6.8	3.62	33.5
Docker	peer 0.org 2.35.102.12.34	208.6	124.0	6.29%	3.16%	6.72	3.5	34.8
Docker	peer 1.org 2.35.102.12.34	207.6	124.0	6.19%	3.16%	6.5	3.53	33.5
Docker	peer 0.org 3.35.102.12.33	204.6	124.0	5.19%	3.16%	6.6	3.62	32.5
Docker	peer 1.org 3.35.102.12.33	66.3	61.1	4.39%	3.15%	6.6	3.59	34.15
Docker	RAFTorderer.35.102.12.32	34.5	24.1	1.51%	0.26%	4.5	9.0	16.1
Docker	ca.org 1.35.102.12.31	6.5	5.8	0.20%	0.00%	729	0	0
Docker	ca.org 2.35.102.12.34	6.5	5.8	0.20%	0.00%	729	0	0
Docker	ca.org 3.35.102.12.33	5.9	5.9	0.29%	0.00%	729	0	0

## Data Availability

The Hyperledger Fabric, Caliper code for implementing and verifying the presented Blockchain-based healthcare design and benchmarking on a multi-hosted testbed protocol are available in a publicly accessible GitHub repository. The prototype code can be found here: https://github.com/niharlipu13/HFEHR_source_code (accessed on: 3 March 2022).
